# Supplementary material for: Parents’ and Guardians’ Willingness to Vaccinate Their Children against COVID-19: A Systematic Review and Meta-Analysis
Source: Vaccines (Basel). 2022 Jan 24;10(2):179. doi: 10.3390/vaccines10020179 (PMC8880569; doi:10.3390/vaccines10020179)
Supplement: Supplementary file 1 [file vaccines-10-00179-s001.zip › Table S1.pdf]

Table S1. Search string used in PubMed.

((("Infant"[Mesh]) OR ((("Child"[Mesh]) OR (children[Title/Abstract])) OR (("Adolescent"[Mesh]) OR ((((((((adolescents[Title/Abstract]) OR (adolescence[Title/Abstract])) OR (teen[Title/Abstract])) OR (teens[Title/Abstract])) OR (teenager[Title/Abstract])) OR (teenagers[Title/Abstract])) OR (youth[Title/Abstract])) OR (youths[Title/Abstract]))))) AND (("COVID-19 Vaccines"[Mesh]) OR (((("SARS-CoV-2"[Mesh]) OR (((((((((((Coronavirus Disease 2019 Virus[Title/Abstract]) OR (2019 Novel Coronavirus[Title/Abstract])) OR (2019 Novel Coronaviruses[Title/Abstract])) OR (SARS-CoV-2 Virus[Title/Abstract])) OR (SARS CoV 2 Virus[Title/Abstract])) OR (SARS-CoV-2 Viruses[Title/Abstract])) OR (2019-nCoV[Title/Abstract])) OR (COVID-19 Virus[Title/Abstract])) OR (COVID 19 Virus[Title/Abstract])) OR (COVID-19 Viruses[Title/Abstract])) OR (SARS Coronavirus 2[Title/Abstract])) OR (Severe Acute Respiratory Syndrome Coronavirus 2[Title/Abstract]))) OR (("COVID-19"[Mesh]) OR ((((((((((((((((((((COVID 19[Title/Abstract]) OR (COVID-19 Virus Disease[Title/Abstract])) OR (COVID 19 Virus Disease[Title/Abstract])) OR (COVID-19 Virus Diseases[Title/Abstract])) OR (COVID-19 Virus Infection[Title/Abstract])) OR (COVID 19 Virus Infection[Title/Abstract])) OR (COVID-19 Virus Infections[Title/Abstract])) OR (2019-nCoV Infection[Title/Abstract])) OR (2019 nCoV Infection[Title/Abstract])) OR (2019-nCoV Infections[Title/Abstract])) OR (Coronavirus Disease-19[Title/Abstract])) OR (Coronavirus Disease 19[Title/Abstract])) OR (2019 Novel Coronavirus Disease[Title/Abstract])) OR (2019 Novel Coronavirus Infection[Title/Abstract])) OR (2019-nCoV Disease[Title/Abstract])) OR (2019 nCoV Disease[Title/Abstract])) OR (2019-nCoV Diseases[Title/Abstract])) OR (Coronavirus Disease 2019[Title/Abstract])) OR (SARS Coronavirus 2 Infection[Title/Abstract])) OR (SARS-CoV-2 Infection[Title/Abstract])) OR (SARS CoV 2 Infection[Title/Abstract])) OR (SARS-CoV-2 Infections[Title/Abstract])))) AND (((("Vaccines"[Mesh]) OR (((((((((((Vaccine[Title/Abstract]) OR (vaccin[Title/Abstract])) OR (vaccins[Title/Abstract])) OR (vaccined[Title/Abstract])) OR (vaccinal[Title/Abstract])) OR (vaccinate[Title/Abstract])) OR (vaccinated[Title/Abstract])) OR (vaccinates[Title/Abstract])) OR (vaccinator[Title/Abstract])) OR (vaccinators[Title/Abstract])) OR (vaccinating[Title/Abstract]))) OR ("Vaccination"[Mesh])) OR (((Vaccinations[Title/Abstract]) OR (Active Immunization[Title/Abstract])) OR (Active Immunizations[Title/Abstract])))))) AND ((("Surveys and Questionnaires"[Mesh]) OR ("Health Surveys"[Mesh])) OR (((((((survey) OR (surveys)) OR (surveyed)) OR (surveying)) OR (questionnaire)) OR (questionnaires)) OR (questionnair)))
