# Supplementary figures and images for: Parents’ and Guardians’ Willingness to Vaccinate Their Children against COVID-19: A Systematic Review and Meta-Analysis
Source: Vaccines (Basel). 2022 Jan 24;10(2):179. doi: 10.3390/vaccines10020179 (PMC8880569; doi:10.3390/vaccines10020179)

**Figure S1. Funnel plot and Egger's test.**

① **Funnel plot:**

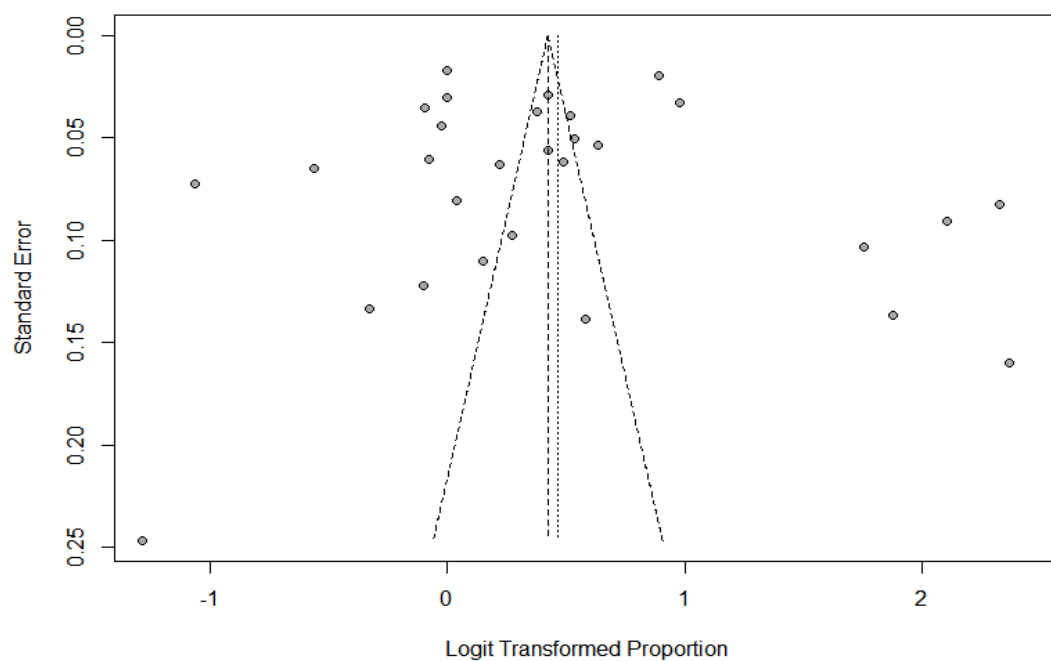

② **Egger's test:  $P = 0.6188 > 0.05$**

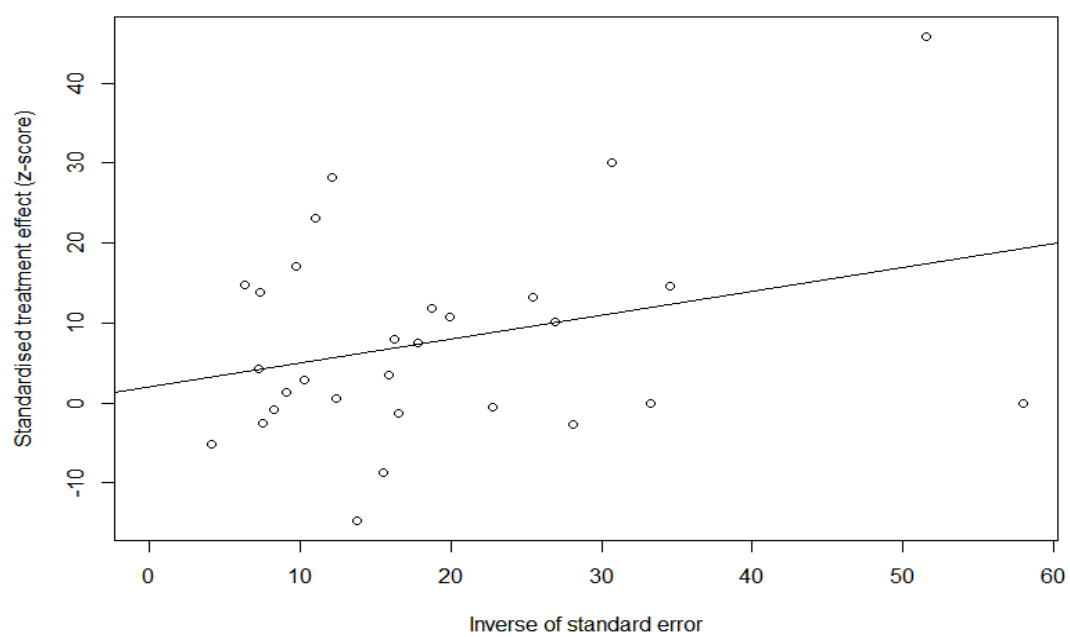

Supplement: Supplementary file 1 [file vaccines-10-00179-s001.zip › Figure S1.pdf]
